# The association between reattempted suicide and incoming calls to the brief contact intervention service, VigilanS: a study of the clinical profile of callers

**DOI:** 10.1186/s12888-022-04503-z

**Published:** 2023-01-09

**Authors:** Jeanne Rusé, Adeline Clenet, Guillaume Vaiva, Christophe Debien, Christophe Arbus, Juliette Salles

**Affiliations:** 1 Department of Psychiatry, University Hospital of Toulouse, CHU Toulouse, Toulouse, France; 2 Centre National de Ressources et de Résilience Lille-Paris, Lille, France; 3 Department of Psychiatry, Centre Hospitalier Universitaire de Lille, Inserm, U1172-LilNCog-Lille Neuroscience and Cognition, Lille, France; 4 Department of Psychiatry, Centre Hospitalier Universitaire de Lille, Lille, France; 5 University Hospital of Toulouse, CHU Toulouse, Department of Psychiatry, Toulouse NeuroImaging Center, ToNIC, University of Toulouse, Inserm, UPS, Toulouse, France; 6 University Hospital of Toulouse, CHU Toulouse, Department of Psychiatry, Infinity (Toulouse Institute for Infectious and Inflammatory Diseases), INSERM UMR1291, CNRS UMR5051, Université Toulouse III, Toulouse, France

**Keywords:** Suicide, VigilanS, Brief contact intervention service, Callers, Borderline personality disorder

## Abstract

**Background:**

Suicide is a major health problem globally. As attempted suicide is a major risk factor for suicide, specific prevention strategies have been designed for use thereafter. An example is the brief contact intervention (BCI). In this regard, France employs a composite BCI, VigilanS, which utilizes three types of contact: phone calls, postcards and a ‘who to contact in a crisis’ card. Previous studies have found that this system is effective at preventing suicide. Nevertheless, VigilanS was not effective in the same way for all the patients included. This observation raises the question of specific adaptation during follow-up for populations that were less receptive to the service. In consideration of this issue, we identified one study which found that incoming calls to the service were linked with a higher risk of suicide reattempts. However, this study did not document the profiles of the patients who made these calls. Better understanding of why this population is more at risk is important in terms of identifying factors that could be targeted to improve follow-up. This research therefore aims to bring together such data.

**Methods:**

We performed a retrospective analysis of 579 patients referred to VigilanS by Toulouse University Hospital (France). We examined the sociodemographics, clinical characteristics, and follow-ups in place and compared the patients who made incoming calls to the service versus those who did not. Subsequently, we conducted a regression analysis using the significantly associated element of patients calling VigilanS. Then, in order to better understand this association, we analyzed the factors, including such calls, that were linked to the risk of suicide reattempts.

**Results:**

We found that 22% of the patients in our sample called the VigilanS service. These individuals: were older, at 41.4 years versus 37.9 years for the non-callers; were more likely to have a borderline personality disorder (BPD) diagnosis (28.9% versus 19.3%); and had a history of suicide attempts (71.9% versus 54.6%). Our analysis confirmed that incoming calls to VigilanS (OR = 2.9) were associated with reattempted suicide, as were BPD (OR = 1.8) and a history of suicide attempts (OR = 1.7).

**Conclusion:**

There was a high risk that the patients calling VigilanS would make another suicide attempt. However, this association was present regardless of the clinical profile. We postulate that this link between incoming calls and reattempted suicide may arise because this form of contact is, in fact, a way in which patients signal that a further attempt will be made.

## Background

Suicide is a major health problem, with the World Health Organization (WHO) reporting that more than 800,000 people die annually worldwide [1]. Suicidal behavior has been identified in all populations, irrespective of age and gender [2]. In France, suicide accounts for 9000 deaths each year, which is one of the highest rates in Europe [2]. Indeed, the number of hospitalization for a suicide attempt increased in the country in the period 2004–2011 (up by 4.8% in men and 2.9% in women) [3]. Moreover, 7.1% of the French population report having made at least one suicide attempt in their lifetime, with 36.8% of this group making more than one.

Attempted suicide is the main risk factor for suicide [4–6]. Specific prevention strategies have therefore been designed for use in this population. An example is the brief contact interventions (BCIs). These maintain the relationship between the providers of mental health care and the patients who have attempted suicide by different interventions. Several BCIs have been studied worldwide including contact through postcards in Australia, Iran, USA and Taiwan [7–11]; telephone interventions in Brazil, China, Iran, Japan, Sweden, Switzerland [12–18] and USA; and crisis cards in United Kingdom [19, 20]. Their effectiveness has been studied extensively in recent years [21], with meta-analyses showing that they reduce the number of suicide attempts per individual [22] and also prevent suicide reattempts at 12 months [23]. The contact modalities employed can comprise: phone calls that focus on a patient’s mental health at the time of the call and their adherence to any post-discharge treatment plan [24]; letters that originate from a medic who met the suicidal patient during a hospital stay [25]; postcards [22] and text messages (SMS), sent as a way to maintain contact [26]; and the provision of resource cards containing telephone number(s) that patients can call to speak to a crisis-management professional [20]. Several researchers have demonstrated the efficacy of these interventions. However, the success of each type of contact has been found to differ in relation to reducing the number of suicide reattempts [12, 27, 28].

Each strategy has its own strengths and limitations (e.g., compared to the other interventions, the cards with numbers to call in a crisis have a more significant effect on those who have made only one suicide attempt). Consequently, the use of a combination of interventions has been suggested to allow for flexible and effective implementation. In this regard, Vaiva and colleagues proposed a composite BCI for France that relies on three types of contact: a phone call for those who have attempted suicide several times; a series of postcards if a patient cannot be reached or has not complied with a post-discharge treatment plan; and a crisis card for first-time attempters [29]. This BCI was first implemented and tested in a French region, and was revealed to be effective [30, 31]. It was therefore extended to the rest of the country, relabeled as VigilanS (Vigilance for the prevention of Suicide recurrence) [32]. This approach has demonstrated a significant degree of efficacy with a sharp decrease in suicide attempts as a function of the service’s penetration, suggesting that a 25% penetrance level would yield a 41% decrease in suicide attempts [33].

Nevertheless, we thought that functioning could be improved particularly by better understanding the characteristics of the patients followed. Indeed, there has been little exploration of the psychiatric diagnoses of the patients followed by VigilanS and while this data is important, some psychiatric diagnoses are associated with increased suicide risk. Moreover, the incidence of suicide reattempts after referrals to the service need also to be specified. Fossi et al. compared the characteristics of these French patients who reattempted suicide to those of the individuals who did not [34]. The authors reported that patients who called VigilanS were associated with a higher risk of reattempting suicide. Nevertheless, Fossi et al. did not describe clinical characteristics including the psychiatric diagnosis of the patients who made incoming calls. This is key data and is required to achieve a more nuanced and better understanding of this finding. Consequently, this study aims to identify the profiles of these individuals in a comparison with other patients who did not use VigilanS. We also analyzed suicide reattempts during follow-up to check the relevance of our results with those from Fossi et al. and to explore the association between suicide reattempt and clinical factors.

## Methods

### Description of the VigilanS service

VigilanS is a six-month monitoring program that is accessed after a suicide attempt. Referrals to the service are made from the emergency department (ED), where the patient is provided with a card containing the telephone number of the VigilanS resource [35]. All patients aged over 18 admitted to the ED for suicide attempt were proposed to benefit from the VigilanS service. From this point on, VigilanS takes charge of any interventions, including scheduled outgoing calls and patient follow-ups over a six-month period. VigilanS has been utilized by Toulouse University Hospital (TUH) since June 2020. The service comprises: a scheduled outgoing call made between days 10 and 21 (D10–21) after a suicide attempt; a hotline operational from Monday to Friday between 10 am and 6 pm (incoming calls); and a scheduled outgoing call in the sixth month, when patients have an end-of-service interview. If the patient is well after 6 months of follow-up, the programed calls are suspended but he or she is still able to call the service if necessary. If the patient is not doing well, the programmed calls are extended. If the service receives no news from the patient, it sends a postcard giving notification of the end of the programmed calls. In terms of incoming calls, we noted from clinical experience that patients call because they have suicidal thoughts or are in pain (anxiety/sadness, anger). In addition, the incoming calls to VigilanS are part of the protection plan carried out during the call at D10 and patients can also call when their health professionals do not answer (busy line, absent referents, etc.).

### Population

TUH provides psychiatric care for the entire population of the city of Toulouse and its environs (totaling 1.2 million people). Patients admitted for suicide attempt systematically benefited from psychiatric assessment: 80% of patients evaluated were referred for Outpatient care and 20% for Inpatient care provided in another establishment. The length of stay in the ED for all patients admitted for suicide attempts is about 30 hours. The patients admitted to the ED for suicide attempt are systematically evaluated by the psychiatric teams. In terms of Outpatient care, patients were referred to their usual follow-up if available or given a referral to a crisis psychiatric ward and some were oriented to their General Practitioner with possible psychiatric support. Patients referred for Inpatient care were admitted to psychiatric wards. From our ED attendance records, we formed a retrospective cohort of 579 patients who had attempted suicide between June 1, 2020 and June 30, 2021 and were referred by the psychiatric team to VigilanS. As some patients might be referred to the service more than once if they reattempted suicide, a single record with all of their referrals was created, retaining the characteristics of the individual at the time of their first contact with the team at VigilanS. Patients who had been referred when they had not made a suicide attempt were excluded.

### Outcomes

The database provided information on the main outcome, i.e., patients making incoming calls to VigilanS, which was a binary variable (yes or no). Two groups were identified: those who made incoming calls (IC), comprising patients who contacted the service by phone at least once during the 6 months of follow up; and those who did not make any incoming calls (the NIC group).

The secondary outcomes were: sociodemographics (age, sex); clinical characteristics (psychiatric diagnosis, past medical history); details of the suicide attempt, including the mode; and subsequent attempt. The subsequent attempt refers to a suicide attempt preceding the one that led to VigilanS inclusion. We found it important to collect this information since it suggests that individuals who had a history of two or more suicidal crises showed a vulnerability to suicidality.

Suicide reattempts were collected in medical files from the TUH. The fact that this hospital is the only one in the city and department to receive patient for suicide attempt enabled us to perform a relevant evaluation of medicalized suicide reattempts. Moreover, all patients admitted to the ED for suicide attempt were evaluated by a psychiatrist. This evaluation helped to distinguish between suicide attempts and non-suicidal self-injury.

### Data collection

The data were collected using the Orbis® software from the databases of TUH (clinical evaluation, suicide reattempts), and VigilanS (call follow-up, suicide reattempts).

### Ethics

The use of the data was approved by the Commission Nationale de l’Information et des Libertés (CNIL), according to the French legislation, MR-004.

### Statistical analysis

The continuous and categorical variables are described in the study using the mean (+/− standard deviation) and/or median (+/− interquartile range), according to their distributions or the numbers and percentages, respectively. Associations between the patients’ categorical characteristics and their diagnoses were tested with the chi-squared or Fischer exact tests (when the expected values were less than 5.0). A multivariable logistic regression model was used to assess links between the patients’ sociodemographics and clinical characteristics and whether they made any calls to VigilanS. The variables identified by the bivariate analyses as significantly associated, with the *p* value set at < 0.05, were included in the initial regression model, along with potential confounding factors relating to the making of an incoming call. We then performed a backwards step-by-step manual selection to produce our final model, controlling for the confounding variables at each stage. We used a regression model in an additional analysis of the suicide reattempt data, specifically to characterize the clinical profile (psychiatric diagnosis) associated with the reattempt. This model included the data that revealed any significant differences in the bivariate analysis. The statistical analyses were performed using the RStudio software, version 1.3.1093©, 2009–2020. Statistical significance was set at *p* < 0.05.

## Results

### Population description

Our sample for the analysis comprised 579 of 580 patients referred to VigilanS between June 1, 2020 and June 30, 2021. The one exclusion was an individual who had not attempted suicide.

The mean age of the cohort was 38.5 years (±16), and 385 (66%) patients were women. Most of the suicide attempts consisted of ingesting drugs (*n* = 502; 86%), hanging (*n* = 23; 4%), cutting (*n* = 21; 4%), jumping (*n* = 19; 3%) and others (*n* = 14; 3%). Forty-two (7%) patients required admission to the intensive care unit (ICU), 172 (37%) were hospitalized on the psychiatric ward and 217 (37%) had positive blood alcohol at the time of ED admission. An adjustment condition had been diagnosed in 134 patients (23%), 199 (34%) with a major depressive disorder (MDD) and 122 (21%) with BPD. Additionally, 334 patients (57%) were non-primary suicide attempters (individuals with a history of more than one attempt) and 105 (18%) made a further attempt in the six-month period of their follow-up with VigilanS. Finally, 347 (72%) patients answered the initial phone call made to them by the service (D10–21) and 317 (66%) the phone contact at 6 months.

### Comparative analysis of the incoming calls

A total of 107 (22%) patients called the VigilanS service (incoming calls). We found significant differences in terms of (IC vs NIC in what follows): age 41.4 years (±16.2) versus 37.9 years (±15.9) (*p* = 0.04); whether an adjustment disorder had been identified *n* = 17 (15.8%) (*p* = 0.03) versus *n* = 117 (24.7%); and the presence of a BPD diagnosis *n* = 31 (28.9%) (*p* = 0.04) versus *n* = 91 (19.3%). Patients in the IC group were more likely to: have a history of suicide attempts *n* = 77 (71.9%) versus *n* = 258 (54.6%) (*p* < 0.01); reattempt suicide *n* = 38 (35.5%) versus *n* = 67 (14.1%) (*p* < 0.01); answer the D10–21 call *n* = 76 (71.0%) versus *n* = 271 (57.4%) (*p* < 0.01); and answer the six-month call *n* = 72 (67.2%) versus *n* = 245 (51.9%) (*p* < 0.01). Additionally, it was more likely that the care team of VigilanS had been able to contact the professionals of those in the IC group *n* = 44 (41.1%) versus *n* = 52 (11.1%) (*p* < 0.01). These patients were also more likely to call the French 911 *n* = 28 (26.1%) versus *n* = 5 (1.05%) (*p* < 0.01).

The complete results are set out in Table 1.

**Table 1:** Characteristics of the patients according to the group

Data n (%) or mean (±SD)	No In. Call *n* = 472	In. Call *n* = 107	*p* value
Sociodemographic Characteristics			
Age (years)	37.9 (±15.9)	41.4 (±16.2)	0.04*
Gender	309 (65.4)	76 (61.2)	0.26
Suicide Attempt Characteristics			
Acute consumption of alcohol	180	37	0.13
Treatment in an ICU	31	11	0.24
Psychiatric inpatient	135	37	0.24
Mode of Suicide Attempt			
Firearm	2 (0.42)	0 (0)	0.15
Drug/poison ingestion	408 (86.4)	93 (86.9)	0.69
Intentional car crash	5 (1.05)	1 (0.93)	0.90
Jumping	14 (2.96)	6 (5.60)	0.43
Drowning	2 (0.42)	0 (0)	0.15
Cutting/injury	19 (4.02)	2 (1.86)	0.17
Hanging/suffocation	19 (4.02)	4 (3.73)	0.88
Other	3 (0.63)	1 (0.93)	0.76
Clinical Characteristics			
Adjustment Disorders	117 (24.7)	17 (15.8)	0.03*
BPD	91 (19.3)	31 (28.9)	0.04*
MDD	160 (33.9)	39 (36.4)	0.07
Alcohol dependence	14 (2.96)	3 (2.80)	0.92
PTSD	5 (1.05)	2 (1.86)	0.56
Suicide Attempt Past Hist.	258 (54.6)	77 (71.9)	< 0.001*
Follow-up Characteristics			
Reattempt	67 (14.1)	38 (35.5)	< 0.001*
Number of reattempts	0.22 (±0.04)	0.79 (±0.74)	< 0.001*
Call Resp. D10-D21	271 (57.4)	76 (71.0)	< 0.001*
Call Resp. M6	245 (51.9)	72 (67.2)	< 0.001*
Postcard sent	250 (52.9)	49 (45.7)	0.18
Call to professionals	52 (11.1)	44 ((41.1)	< 0.001*
Call to French 911	5 (1.05)	28 (26.1)	< 0.001*
Call to relatives	23 (4.87)	13 (12.1)	0.07
Support available	443 (93.8)	96 (89.7)	0.19

*SD* standard deviation, *In. Call* incoming call, *No In. Call* no incoming call, *ICU* intensive care unit, *BPD* borderline personality disorder, *MDD* major depressive disorder, *PTSD* post-traumatic stress disorder, *Suicide Attempt Past Hist.* suicide attempt past history, *Call Resp. D10-D21* response to the call made between days 10 and 21, *Call Resp. M6* response to the call made at 6 months

* denotes significant result (*p* value < 0.05)

### Multivariable logistic regression

The regression model found that BPD (OR = 1.8; 95% CI = [1.02–3.06]), age (OR = 1.01, 95% CI = [1.01–1.03]) and a history of suicide attempts (OR = 1.9, 95% CI = [1.2–3.05]) were all associated with the IC group. The diagnosis of an adjustment disorder was not, however, linked to incoming calls in this model (*p* = 0.22).

### Reattempts

An analysis was conducted of the clinical diagnosis associated with any suicide reattempts in the 6 months following the initial act. We found that a BPD diagnosis was more common in the patients who reattempted suicide 35% versus 17% for those not diagnosed with the condition (*p* < 0.01). Conversely, the identification of an adjustment disorder was more frequent in the no-reattempt group (12% versus 25%; *p* < 0.01). We also discovered that suicide was reattempted more often by women (20% versus 15%, *p* < 0.01) and patients with a history of attempts (73% versus 54%; *p* < 0.01). The regression model based on the significant results from the bivariate analysis found that a BPD diagnosis (OR = 1.8; 95% CI = [1.1–3.0]), a history of attempting suicide (OR = 1.7; 95% CI = [1.1–2.9]), and incoming calls to VigilanS (OR = 2.9; 95% CI = [1.7–4.6]) were all associated with reattempts.

**Fig. 1:**
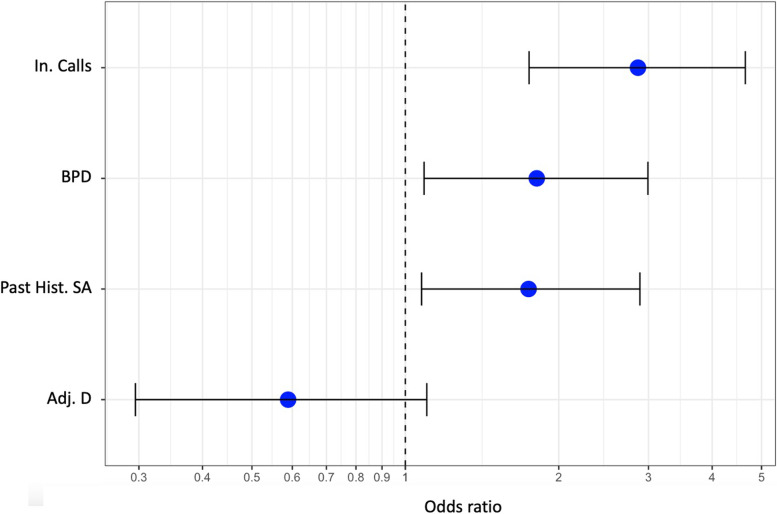
Representation of the odds ratios of the regression model, with an independent value for suicide reattempts. Adj. D: adjustment disorder; BPD: borderline personality disorder; Past Hist. SA: past history of suicide attempt

The complete analysis is presented in Table 2 and Fig. 1.

**Table 2:** Results of the regression analysis for the independent value of suicide reattempts

	OR	2.5%	97.5%	*p* value
(Intercept)	0.11	0.07	0.17	2.2e-16
Incoming calls	2.86	1.74	4.64	2.375e-05*
Adj. D	0.58	0.29	1.10	0.11
BPD	1.81	1.08	2.99	0.02*
Past Hist. SA	1.74	1.07	2.88	0.02*

*Adj. D* adjustment disorder, *BPD* borderline personality disorder, *Past Hist. SA* past history of suicide attempts

*denotes significant result (*p* value < 0.05)

## Discussion

We aimed to investigate the profiles of patients who made a phone call to the BCI-based VigilanS service after they were referred following a suicide attempt. The cohort that we studied was 66% female and had a mean age of about 40. This reflects the characteristics previously described in other studies on those who attempt suicide [36, 37]. The main mode utilized by our sample was the ingestion of drugs, which is also consistent with previous reports [38, 39]. However, while 45% of relevant studies described a wide range of alcohol consumption (10 to 73%), a meta-analysis in 2004 identified a mean of 40% [40], which is again similar to earlier reports from our hospital [41]. The figure for hospitalization was 36%, which is higher than previously described in Spain (26.8%) [42] and Finland (24%) [43], but lower than in Switzerland and England [30, 31]. It is also below the figure of almost 50% contained in the WHO/Euro Multicentre Study on Parasuicide [24]. The number of patients admitted to our ICU is comparable with previous reports [44]. The reattempt rate in our cohort was 18%, which is higher than in the research by Fossi et al., where it was 8%. It may be that this is due to a lower rate of non-primary suicide attempters in our cohort (57%) versus theirs (47%). Nevertheless, the 18% reattempt value that we uncovered is comparable with previous observations that have identified rates of 14 to 25% for patients treated at medical emergency units [45–50]. The data collected from the ED could, however, be incomplete, as Jollant et al. found that 39.3% of suicide attempters do not present at hospital [51]. We wondered whether the higher reattempt rate in our study versus that by Fossi et al. could also be explained by more contact with patients by VigilanS, enabling better monitoring of any reattempts. Indeed, in our cohort, the success rate for contact was about 60% at 6 months, compared to about 20% in Fossi. The percentage of patients who made incoming calls was also higher in our study (22% versus 3.5%).

We also found that the patients who called VigilanS were more likely to be older, have a BPD diagnosis and a history of suicide attempts. This indicates that the profile of those who are at significant risk of suicide encompasses a suicide-attempt background [4–6]. Additionally, a BPD diagnosis is associated with recurring suicidal behavior, gestures, threats, or self-harm [52]. Moreover, BPD patients have: a mean of three lifetime suicide attempts [53, 54]; treatment histories marked by multiple failed attempts; frequent attendances at emergency rooms; and hospitalizations for attempting suicide and threats of suicide [55]. Finally, suicide is more common among patients with BPD than in the general population [56, 57]. These findings are interesting, since they suggest that VigilanS should be able to clearly identify who is part of this high-risk population. They also point to a possible need for specific training on interventions that reduce suicide in the BPD population, i.e., the GPM approach [58].

Similar to Fossi et al. [34], we found that incoming calls were associated with suicide reattempts. Intriguingly, the making of calls to VigilanS was significantly associated with the reattempt risk, irrespective of whether there was a BPD diagnosis and/or a history of attempts, demonstrating a specific effect on the suicide-reattempt risk in those who make such calls. We have noted in clinical practice that some patients rang VigilanS to report a suicide attempt, leading to rescues being initiated by the team. This reality on the ground is highlighted in our research by the significant association between incoming calls and calls to the French 911 and other professionals. It could therefore first be hypothesized that incoming calls are linked to suicide attempts because they enable better detection and medicalization. Second, it is also possible that these calls lead to a decrease in the severity and consequences of an attempt, although these are currently only hypotheses with no corroborating data.

Despite the interesting results described in this paper, our research has some limitations, including: 1) the retrospective design and absence of new data; 2) the possible imprecision of the BPD diagnoses extracted from the CIM-10 codings; 3) the cross-sectional design, which only enabled us to identify associations and not test the explicative hypotheses; and 4) the single-site model employed. Finally, this sample was selected during the COVID-19 pandemic which could have had some impact on the interpretation of the results. Indeed, we observed an increased amount of referrals for suicide ideation and suicide attempts in 2021 at our ED (+ 30% versus the year 2019). The higher number of referrals mainly concerned adolescents or young adult patients. Only young adults (over 18 years old) were directed to VigilanS and we hope that the influence of the pandemic was limited on the care proposed. In fact, the service remained operational during this period and every patient over the age of 18 was able to benefit from it.

In conclusion, the patients who made incoming calls to VigilanS are part of a cohort at high risk of reattempting suicide. This demonstrates clearly that the service is being utilized by the targeted group. Meanwhile, incoming calls were associated with more reattempts and rescues, irrespective of our population’s characteristics, suggesting that the system could contribute to both improving the identification of those who may try to kill themselves and the provision of better care.

## Data Availability

The datasets used during the study are available from the corresponding author on reasonable request.

## Abbreviations

BCI: Brief contact intervention
BPD: Borderline personality disorder
ED: Emergency department
IC: Incoming call
ICU: Intensive care unit
MDD: Major depressive disorder
NIC: Non-incoming call
TUH: Toulouse University hospital
WHO: World Health Organization
